# Metabolic Reconstruction for Metagenomic Data and Its Application to the Human Microbiome

**DOI:** 10.1371/journal.pcbi.1002358

**Published:** 2012-06-13

**Authors:** Sahar Abubucker, Nicola Segata, Johannes Goll, Alyxandria M. Schubert, Jacques Izard, Brandi L. Cantarel, Beltran Rodriguez-Mueller, Jeremy Zucker, Mathangi Thiagarajan, Bernard Henrissat, Owen White, Scott T. Kelley, Barbara Methé, Patrick D. Schloss, Dirk Gevers, Makedonka Mitreva, Curtis Huttenhower

**Affiliations:** 1 The Genome Institute, Washington University School of Medicine, St. Louis, Missouri, United States of America; 2 Department of Biostatistics, Harvard School of Public Health, Boston, Massachusetts, United States of America; 3 J. Craig Venter Institute, Rockville, Maryland, United States of America; 4 Department of Microbiology and Immunology, University of Michigan, Ann Arbor, Michigan, United States of America; 5 Department of Molecular Genetics, Forsyth Institute, Cambridge, Massachusetts, United States of America; 6 Department of Oral Medicine, Infection and Immunity, Harvard School of Dental Medicine, Boston, Massachusetts, United States of America; 7 Institute for Genome Sciences, University of Maryland School of Medicine, Baltimore, Maryland, United States of America; 8 The Broad Institute of MIT and Harvard, Cambridge, Massachusetts, United States of America; 9 Architecture et Fonction des Macromolécules Biologiques, UMR 6098 CNRS, Université de la Méditerranée, Marseille, France; 10 Biology Department, San Diego State University, San Diego, California, United States of America; University of California Davis, United States of America

## Abstract

Microbial communities carry out the majority of the biochemical activity on the planet, and they play integral roles in processes including metabolism and immune homeostasis in the human microbiome. Shotgun sequencing of such communities' metagenomes provides information complementary to organismal abundances from taxonomic markers, but the resulting data typically comprise short reads from hundreds of different organisms and are at best challenging to assemble comparably to single-organism genomes. Here, we describe an alternative approach to infer the functional and metabolic potential of a microbial community metagenome. We determined the gene families and pathways present or absent within a community, as well as their relative abundances, directly from short sequence reads. We validated this methodology using a collection of synthetic metagenomes, recovering the presence and abundance both of large pathways and of small functional modules with high accuracy. We subsequently applied this method, HUMAnN, to the microbial communities of 649 metagenomes drawn from seven primary body sites on 102 individuals as part of the Human Microbiome Project (HMP). This provided a means to compare functional diversity and organismal ecology in the human microbiome, and we determined a core of 24 ubiquitously present modules. Core pathways were often implemented by different enzyme families within different body sites, and 168 functional modules and 196 metabolic pathways varied in metagenomic abundance specifically to one or more niches within the microbiome. These included glycosaminoglycan degradation in the gut, as well as phosphate and amino acid transport linked to host phenotype (vaginal pH) in the posterior fornix. An implementation of our methodology is available at http://huttenhower.sph.harvard.edu/humann. This provides a means to accurately and efficiently characterize microbial metabolic pathways and functional modules directly from high-throughput sequencing reads, enabling the determination of community roles in the HMP cohort and in future metagenomic studies.

## Author Summary

The human body is inhabited by trillions of bacteria and other microbes, which have recently been studied in many different habitats (including gut, mouth, skin, and urogenital) by the Human Microbiome Project (HMP). These microbial communities were assayed using high-throughput DNA sequencing, but it can be challenging to determine their biological functions based solely on the resulting short sequences. To reconstruct the metabolic activities of such communities, we have developed HUMAnN, a method to accurately infer community function directly from short DNA reads. The method's accuracy was validated using a collection of synthetic microbial communities. Applying HUMAnN to data from the HMP, we showed that, unlike individual microbial species, many metabolic processes were present among all body habitats. However, the frequencies of these processes varied dramatically, and some were highly enriched within individual habitats to provide niche specialization (e.g. in the gut, which is abundant in food matter but low in oxygen). Other community functions were linked specifically to properties of the human host, such as biochemical processes only present in vaginal habitats with particularly high or low pH. Studying additional environmental or disease-associated communities using HUMAnN will further improve our understanding of how the microbial organisms in a community are linked to the biological processes they carry out.

## Introduction

Human-associated microbial communities interact directly with their hosts by means of metabolic products and immune modulation, and environmental communities are further responsible for a wide range of biochemical activities [1]. Metagenomic sequencing provides a culture-independent means of studying these diverse microbiota within different ecological niches, including sites in the human body that differ strikingly in microbial composition and subsequent impacts on health [2], [3], [4]. The gut microbiota in particular have been shown to play an important role in host metabolism [5], [6] and immune response [4], and mechanisms of commensal microbial contribution to disease have been established e.g. in the vaginal [7] and skin [8] communities as well. These studies have demonstrated the importance of assaying microbial pathways, metabolism, and individual gene products by means of metagenomic sequencing to determine their roles in community-wide interactions and phenotypes. A functional interpretation of metagenomic sequences is thus key to connecting the metabolic and functional potential of a microbial community with its organismal population structure and with its influence on the surrounding environment or human host.

**Figure 1:**
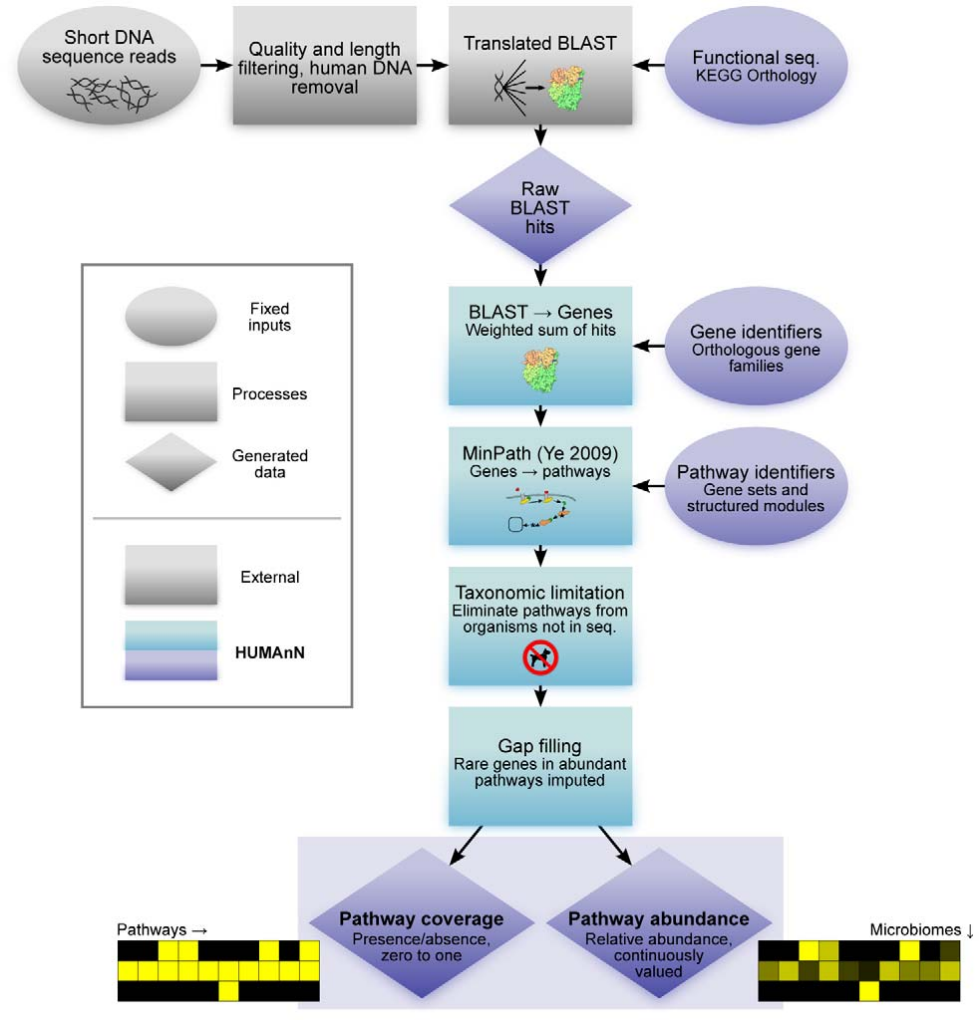
Overview of the HUMAnN method for metabolic and functional reconstruction from metagenomic data. The HMP Unified Metabolic Analysis Network (HUMAnN) software recovers the presence, absence, and abundance of microbial gene families and pathways from metagenomic data. Cleaned short DNA reads are aligned to the KEGG Orthology [26] (or any other characterized sequence database) using accelerated translated BLAST. Gene family abundances are calculated as weighted sums of the alignments from each read, normalized by gene length and alignment quality. Pathway reconstruction is performed using a maximum parsimony approach followed by taxonomic limitation (to remove false positive pathway identifications) and gap filling (to account for rare genes in abundant pathways). The resulting output is a set of matrices of pathway coverages (presence/absence) and abundances, as analyzed here for the seven primary body sites of the Human Microbiome Project.

The functions of human-associated microbes are of particular interest, and the Human Microbiome Project (HMP [9]) has thus performed a comprehensive study of microbial communities from many different body sites in a reference population of disease-free adult subjects. The study's metagenomic data comprise over 3.5 Tbp of shotgun DNA sequences drawn from seven body habitats (including oral, gut, urogenital, nasal, and skin) from over 100 individuals. Using these data, we sought to address questions pertaining specifically to microbiome function: what metabolic and broader biomolecular functions are present within the human microbiome, how do they provide specialization within the microbial niches of distinct body sites, and how do they vary across the human host population? To address these in a high-throughput manner, we have developed a scalable methodology to reconstruct the functional potential of microbial communities from metagenomic sequences, the HMP Unified Metabolic Analysis Network (HUMAnN). To avoid the need for assembly of metagenomic reads, HUMAnN (Figure 1) allows direct profiling of the metabolic potential of microbial communities as represented by orthologous gene family and pathway abundances. The computational methodology incorporates a series of gene- and pathway-level quantification, noise reduction, and smoothing steps in order A) to identify which pathways are present or absent within a metagenomically sequenced community and B) to determine their relative abundances. HUMAnN's predictive accuracy was validated quantitatively using data from four synthetic communities, and it was subsequently used to characterize metabolic function throughout the human microbiome.

Most analytical methods for microbial community assays focus on organismal membership and population structure, i.e. “who's there” in a community [5], [10]. However, functional characterization of community metagenomes is additionally necessary to determine what metabolism and other biological activity may be occurring [11], [12]. This presents a distinct set of challenges, since inter-dependent organisms within a community may share many functional components in addition to playing individually specialized roles. Current metagenomic approaches for characterizing microbial community function include IMG/M [13], MG-RAST [14], and the recently expanded MEGAN tool [15]. Each of these relies on a “best-BLAST-hit” approach, in which individual short reads from a sequenced community (or open reading frames from assembled DNA) are searched against a characterized reference database using translated BLAST. This approach has been used to show the importance of specific community metabolic processes in a range of environmental ecologies, including ocean water and the human gut [16]. Gianoulis et al [17] in particular found that in a collection of 37 ocean communities, metabolic differences correlated specifically with environmental features such as temperature, depth, and salinity. In a particularly large dataset of 124 gut metagenomes, the MetaHIT consortium [11] qualitatively identified metabolic pathways using genes predicted from assembled sequences, which were subsequently suggested to be associated with host phenotypes including obesity [12]. Several additional studies have shown the importance of testing for pathways differentially abundant among communities of interest, e.g. among ocean environments [18] or within the infant gut [19], [20]. However, although each of these results demonstrates the importance of community metabolism and function, no one method has yet been quantitatively evaluated as a means of reconstructing microbial pathway abundances from metagenomic data.

In order to determine the distribution of microbial function within the human microbiome, we thus first validated HUMAnN's ability to quantify metabolic pathway abundances in four synthetic metagenomes containing up to 100 organisms. These were recovered with correlations over 0.9, consistently outperforming best-BLAST-hit approaches. We proceeded to scale our analysis to perform metabolic reconstruction on 649 human microbiome samples drawn from the buccal mucosa, supragingival plaque, and tongue dorsum (oral sites), anterior nares (nasal), retroauricular crease (skin), and stool (gut) communities from 102 individuals. We identified 196 metabolic pathways and 168 small modules that were differentially abundant among body sites, and we highlight here associations with environmental pH and enrichment for glycosaminoglycan degradation as examples from the vaginal and gut communities, respectively. Metabolic module abundances were substantially more variable among body sites and among individuals than was module coverage, indicating a connection between selective pressures in each microbial niche and the pathways carried by members of the community. Finally, as HUMAnN simultaneously reconstructs large pathways, specific metabolic modules, and individual enzymatic gene families, we discuss an example of glutamate metabolism in the gut community as it interacts with specific carbohydrate active enzymes (CAZys [21]). An implementation of HUMAnN is publicly available at http://huttenhower.sph.harvard.edu/humann, and the methodology can be equivalently applied to metatranscriptomic or metaproteomic data using any gene or pathway catalog of interest for future studies.

## Methods

Here, we describe the methodology employed in this study in two parts: first, the computational pipeline for metagenomic metabolic reconstruction implemented in HUMAnN, and second its application to the 741 microbial community samples of the Human Microbiome Project. HUMAnN inputs metagenomic DNA sequences and infers community-wide gene and pathway abundances through a process of seven steps (Figure 1):

1. Short reads are sequenced from a community sample, quality and length filtered, and screened for residual host (human) DNA. This process is carried out by the user externally to HUMAnN.
2. Reads are searched against a characterized protein sequence database. HUMAnN can operate using results from several standard or accelerated translated BLAST implementations and from different orthologous protein family catalogs; for the HMP we employed MBLASTX and the KEGG Orthology (see below).
3. For each metagenomic sample, HUMAnN recovers the abundances of individual orthologous gene families by counting its reads' BLAST hits in a weighted manner, normalized by each gene family's average sequence length.
4. Genes are assigned to pathways using MinPath [22], a maximum parsimony approach to explaining observed genes with available pathways.
5. Pathways unlikely to be present based on the BLAST hits' approximate organismal profiles are removed in a taxonomic limitation step, which also allows normalization for genes' average copy number.
6. A biological smoothing or gap filling step is performed, preventing small numbers of apparently absent genes in an otherwise abundant pathway from diminishing its presence due to noise.
7. Finally, HUMAnN assigns each pathway a coverage (presence/absence) score in each sample based on the detection of all of its constituent genes, as well as an abundance score indicating its relative abundance in the sample's metagenome.

HUMAnN has additionally adapted ecological diversity metrics in order to provide functional diversity and richness profiles for each sample, and we validated its gene- and pathway-level accuracy using four synthetic communities of varying complexity.

To assess microbial community function and metabolism in the human microbiome, we applied this process to the metagenomic data generated by the HMP [9], comprising >3.5 Tbp of microbial DNA from 7 body sites spanning 102 individuals (Table 1). We identified modules over- or under-represented in individual body sites using the LEfSe [23] biomarker detection system, as well as associating the resulting gene and module abundances with subject clinical metadata and with external data including CAZy [21] abundances using standard nonparametric Spearman correlation.

**Table 1:** Metagenomic samples, functional modules, and metabolic pathways analyzed here and differentially abundant in the human microbiome.

		Ret. crease		Stool		Anterior nares		Posterior fornix		Sup. plaque		Buccal mucosa		Tongue dorsum		Total
**Samples**		26		136		87		53		115		109		123		649
	*Total*	215		230		198		180		223		203		217		232
**Modules**	*Abd. ±*	46	6	33	26	11	6	25	107	28	18	14	3	11	2	168
	*Cov. ±*	11	1	11	2	0	21	14	1	0	8	2	4	7	1	24
	*Total*	288		290		290		275		291		291		291		297
**Pathways**	*Abd. ±*	55	23	28	24	27	4	38	109	24	8	16	22	8	6	196
	*Cov. ±*	31	4	29	5	0	39	30	7	0	15	18	8	9	12	41

649 total microbiome samples spanning seven body habitats were reconstructed into 232 small functional modules and 297 large pathways using HUMAnN. Differential abundance (over- or under-enrichment) in at least one body site was tested using LEfSe [23] and differential coverage by a minimum prevalence (90% for modules, 50% for pathways) threshold.

### Metagenomic short read preprocessing

The filtering criteria applied to HMP short reads are representative of HUMAnN's sequence preprocessing requirements. As fully described elsewhere by the HMP [24], 100 bp paired-end Illumina shotgun metagenomic reads were screened for duplicate reads and for residual human sequences. BWA [25] trimming was then applied at q = 2, followed by low-complexity filtering, and sequences resulting in less than 60 remaining valid bases were discarded. In practice, any steps removing non-microbial DNA and low-quality reads should be sufficient for HUMAnN, as retained uncharacterizable reads will be removed during the subsequent BLAST search.

### Translated BLAST against characterized protein sequences

Sequences passing preprocessing criteria are then searched against a characterized protein sequence database. For the HMP, we employed MBLASTX (MulticoreWare, St. Louis, MO), an accelerated translated BLAST implementation, with default parameters against a functional sequence database including the KEGG Orthology v54 [26]. All 741 HMP samples were searched in less than 13,000 CPU-hours (with 32 GB memory required on average), resulting in an average of 36% of reads mapped to at least one orthologous family, and up to the 20 most significant hits at E<1 were retained and used for further processing. The HUMAnN software additionally includes support for NCBI BLASTX, USEARCH [27], and MAPX (Real Time Genomics, San Francisco, CA) and has been tested with other sequence databases including MetaCyc [28] and CAZy [21].

### Orthologous gene family abundances

HUMAnN next summarizes these BLAST results as the number of reads that matched each protein family, weighted by the quality of the matches. We used KEGG Orthology gene families (KOs) as defined by KEGG [26], a catalog of organism-independent identifiers corresponding to groups of gene sequences carrying out comparable biochemical functions. For our analysis, each KO *i* consists of a set of one or more specific gene sequences *G_i_* = {*g_i_*_,1_, *g_i_*_,2_, …} from individual organisms annotated in KEGG v54. Orthologous family abundances *w_i_* were calculated independently within each metagenome for KO *i* and read *j* as:
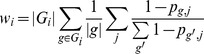
where |*g*| is the nucleotide length of gene sequence *g* in KO *i*, |*G_i_*| is the number of such sequences, and *p_i,j_* is the p-value of the MBLASTX hit of read *j* to sequence *g* (or 1 if no such hit occurred), calculated from the E-value as *p* = 1-e^−*E*^. That is, the relative abundance of KO *i* in a metagenome is the number of reads *j* that map to a gene sequence in the family, weighted by the inverse p-value of each mapping and normalized by the average length of all gene sequences in the orthologous family. Comparisons among alternative weighting schemes (bit score, inverse E-value, and sigmoid-transformed E-value) suggested that the specific method by which multiple BLAST hits were combined had little effect on outcome (Supplemental Figure S3). Although it was surprisingly unnecessary to normalize by average gene family length in order to recover accurate pathway abundances (Supplemental Figure S1), this step was critical in inferring accurate gene family abundances (Supplemental Figures S3–4).

### Assigning gene families to pathways and modules

For each sample, the process above assigns each KO family a relative abundance; KOs are then consolidated into one or more pathways (or modules) using MinPath [22]. MinPath defines each pathway as an unstructured gene set and selects the fewest pathways that can explain the genes observed within each community. More specifically, HUMAnN associates each KO family *i* with a vector of relative abundances *w* = [*w_i_*_1_, *w_i_*_2_, …] in each metagenome. For the HMP, KOs were then assigned to zero or more pathways and modules (both as defined in KEGG) using MinPath v1.2 [22]. KOs assigned to two or more pathways/modules are effectively duplicated and their abundance included in each; this results in two independent vectors of abundance tuples of the form (KO, pathway ID) and (KO, module ID) for each metagenome. More formally, each sample is at this stage represented as a vector *w^p^* = [*w_j_*_1,*p*1_, *w_i_*_1,*p*2_, *w_i_*_2,*p*1_, *w_i_*_2,*p*2_, …], where *w_i,p_* = *w_j_* for all pathways *p*, and an analogous *w^m^* for modules.

For evaluation purposes, best-BLAST-hit pathway and module assignments in Supplemental Figures S1–3 were performed using only best BLAST hits, without weighting by quality of hit, normalization by gene sequence length, maximum parsimony assignments by MinPath, or the additional HUMAnN steps described below. Each best-BLAST-hit was counted once and duplicated, as for HUMAnN, into all pathways or modules within which the targeted gene occurred. For additional evaluations with best-BLAST-hit in combination with other HUMAnN processing steps, see Supplemental Figure S4.

### Filtering pathways by taxonomic limitation with copy number normalization

We found an additional module/pathway filter step to be useful in removing false positive pathways selected by MinPath. Specifically, by retaining a very approximate organismal abundance profile of gene families hit during the initial BLAST process, HUMAnN is able to remove pathways in gross disagreement with observed taxa in an unsupervised manner. This is performed leniently in order to be minimally disruptive in e.g. microbial communities rich in uncharacterized organisms, and often results in depletion of false positive metazoan pathways. Specifically, taxonomic limitation is performed by removing only (KO, ID) tuples for which the same KO was assigned to multiple pathways or modules. For each sample, approximate abundances for each organism *o* in KEGG were calculated as a sum over all weighted, normalized BLAST hits to sequences from that organism:
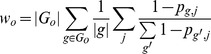
Each pathway/module was then assigned an approximate expected relative abundance by summing *w_o_* values over all organisms' genomes in which it was annotated. Finally, any (KO, ID) pair with two or more IDs *and* corresponding to a pathway/module with observed relative abundance below the average expected abundance for that ID was removed. That is, for *δ_o,p_* = 1 if pathway *p* was annotated to organism *o* in KEGG and 0 otherwise, a pathway's expected abundance was:
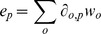
and all *w_i,p_*_1_ such that *w_i,p_*_2_>*w_i,p_*_1_>0 and 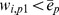 were set to zero. Median and inter-quartile range cutoffs were also evaluated for this limitation process and the settings described here were retained due to optimal performance on synthetic data (see below and Supplemental Figure S1).

When performing this step, HUMAnN can additionally use the data on approximate taxonomic composition to divide each gene's abundance by its expected copy number in the detected organisms, providing a degree of additional normalization (as gene family copy number should not influence the abundance of pathways in which they're carried). This contrast is reflected in Supplemental Figure S1 as “Tax” and “TaxC,” respectively. As shown by our evaluation, the taxonomic limitation process both with and without copy number normalization substantially reduced false positive pathway detections caused by gene families that participate in multiple processes.

### Smoothing pathways by gap filling

Taxonomic limitation was used by HUMAnN to reduce false positive pathways, and we found a small degree of replacement or “gap filling” of certain missing genes to likewise reduce false negatives. A small number of low abundance genes within otherwise abundant pathways often occurred due to noise or poor BLAST hits. Biological gap filling was added to increase the effective contribution of unobserved members of otherwise abundant pathways, although this had a minimal impact on overall performance in most communities. Within each retained pathway/module ID, KOs with relative abundance 1.5 interquartile ranges below the pathway median were boosted to an effective abundance equal to median for purposes of subsequent calculations. That is, for all pathways *p* such that there existed some *w_i,p_*>0, let 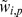 be the lower inner fence of *w_i,p_* over all *i*∈*p*, and each *w_i,p_* for *i*∈*p* was set to max(*w_i,p_*, 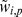). Add-one and Witten-Bell smoothing [29] were also evaluated as alternative methods for gap filling independent of prior biological knowledge; add-one replaces missing genes in abundant pathways with a constant value, and Witten-Bell replaces missing genes sample-wide with a small probability mass estimated from abundant genes. However, neither was retained due to a lack of improvement on synthetic data (Supplemental Figure S1).

### Pathway/module coverage and abundance

The final outputs for each sample were thus coverage (presence/absence) and abundance values for KEGG modules and pathways. These two types of entities are quantified somewhat differently by HUMAnN, but with equivalent semantics. Pathways are defined as unordered sets of orthologous gene families; modules are defined by KEGG as combinations of required, optional, or complementary genes in notation resembling conjunctive normal form. In both cases, coverage is calculated to indicate the likelihood that all genes needed to operate the pathway or module are present; abundance is calculated as the average copy number of the pathway or module's operational subset. The definition of “operation” changes since pathways, as unordered sets, are assumed to include redundant genes (which are not explicitly indicated), whereas alternative means of accomplishing a specific metabolic module can be explicitly taken into account.

Thus each pathway's coverage and abundance were calculated fairly simply. Given the vector *w^p^*, coverage for each pathway *p* in a sample was calculated as the fraction of KOs in the pathway that were confidently present, specifically with abundance greater than the overall sample median. That is:
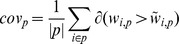
Pathway abundance was calculated as the average of the upper half of its individual gene abundances, in order to be robust to low-abundance alternative enzymes; that is:
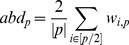
for [*p*/2] the most abundant half of *w_i,p_*.

Modules were determined to be covered only if each gene in at least one path satisfying the module was confidently present. Specifically, given the vector *w^m^*, coverage for each module *m* in a sample was calculated as the harmonic mean of the Χ^2^ CDF with 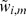 degrees of freedom evaluated at *w_i,m_* for each required *i*∈*m*, maximizing over optional genes *i* and alternative submodules. That is, the probability of each gene family *i* being present in pathway *p* by chance was assigned based on the sample-wide median abundance 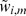 (thus adjusting for sequencing depth). This has the effect of strongly penalizing low-abundance genes, i.e. a module could not be present without all its constituent required gene abundances being confidently nonzero. Module abundances were calculated more simply as the harmonic mean of the sample gene family abundances *w_i, m_*, replacing pathways' arithmetic means since alternative enzymes are explicitly known and taken into account. These choices of parameterization both for pathways and for modules were again validated using multiple synthetic communities (Supplemental Figure S1).

### Tests for significant associations with subject phenotype and sample metadata

Three classes of statistical tests were used to assess metabolic variability across the human microbiome. First, pathways and modules differentially abundant in at least one of the seven analyzed body sites were determined by the LEfSe system for metagenomic biomarker discovery [23]. These differences were summarized into overall patterns of variation using principal component analysis on a matrix of average module abundances per body site, Winsorized at 20% (a robust arithmetic mean [30]), filtered at a minimum of 0.01% in at least one site, and normalized to z-scores. Since LEfSe is not appropriate for HUMAnN's binary pathway coverage scores, we determined site-enriched or underenriched pathways and modules as follows: a module was in aggregate present at a site if it occurred with coverage ≥0.9 in ≥90% of the site's samples; absent if it occurred with coverage ≤0.1 in ≥90% of samples; and differential if it was present in at least one site and absent in at least one other. Pathways were analyzed identically using a ≥0.5 coverage criterion, since no large pathways consistently had coverage ≥0.9.

The third test described here associated pathway and module abundance not with human microbiome body sites, but with one or more of the subject clinical metadata variables described by the HMP [9]. These included continuous descriptors of each sample (e.g. subject age, body mass index, vaginal introitus and posterior fornix pH for women, etc.) as well as categorical variables (e.g. gender or location, see Supplemental Table S1). Pathway and module abundances were associated with these metadata first by stratifying by body site. Within each body site, each pathway/metadata pair present above 0.01% in at least 10% of samples was independently tested using Spearman's ρ for continuous metadata and the Kruskal-Wallis nonparametric ANOVA for categorical, after removing any outliers outside of the upper or lower inner fences. The resulting p-values were corrected using the Benjamini-Hochberg method within each body site and thresholded at a minimum FDR q<0.1.

### Synthetic mock communities for validation

Four *in silico* synthetic communities were constructed to validate parameter choices and to determine HUMAnN's predictive accuracy. Inspired by Mavromatis et al [31], we generated four communities, two of low complexity (LC, 20 organisms) and two of high (HC, 100 organisms). One HC and one LC had even distributions with all organisms at equal abundance, and the remaining two had log-normally distributed random abundances (see Supplemental Table S2). Organisms for the LC communities were manually selected from KEGG v54 curated reference genomes associated with the human microbiome, and HC communities were randomly generated from all manually curated bacterial genomes. A MAQ [6] error model was constructed using one lane of Illumina reads and quality scores; 10^6^ synthetic reads were generated from this error model per organism. These were randomly mixed in proportion to organismal abundances to a total of 10^6^ 100 bp reads per community. These synthetic reads were BLASTed as above, with any hits at >90% identity discarded so as to prevent overestimates of accuracy based solely on well-characterized genomes.

Finally, gold standards of pathway/module coverage and abundance were constructed for each community by listing A) the pathways/modules annotated to at least one organism in the community and B) multiplying these by the organisms' abundances, respectively. Inferred pathway and module coverages and abundances were also calculated by applying HUMAnN to these synthetic reads as described above for the HMP samples. All software implementing these processes and the specific error model, synthetic communities, and data used for HUMAnN in the HMP are available at http://huttenhower.sph.harvard.edu/humann.

### Data and availability

All metabolic reconstructions generated by this study are publicly available at http://hmpdacc.org/HMMRC. Taxonomic abundances derived from shotgun data are provided at http://hmpdacc.org/HMSCP, and input Illumina reads at http://hmpdacc.org/HMIWGS. The open source HUMAnN software can be obtained at http://huttenhower.sph.harvard.edu/humann.

## Results

We first validated HUMAnN's ability to accurately quantify microbial community function using a collection of four synthetic metagenomes. We proceeded to reconstruct metabolic pathways and modules for 741 human microbiome samples comprising a total of 3.5 Tbp of sequence from 18 body habitats from 102 subjects assayed metagenomically by the HMP. 686 of these samples passed quality control (see [9]), and after grouping bilateral habitats (left and right retroauricular creases), seven habitats included at least 25 samples: buccal mucosa, supragingival plaque, and tongue dorsum in the oropharynx; anterior nares and retroauricular crease representing airways and skin; stool samples representing the gut; and the vaginal posterior fornix. These habitats together comprised the 649 total microbial community samples analyzed here (Table 1). In the following sections, we report on their metabolic reconstructions using modules and pathways, discuss instances of inter- and intra-habitat functional variation, and present examples relating community function to microbial abundances and to host phenotype.

### Accurate pathway coverage and abundance reconstructions from short DNA reads assessed in synthetic communities

To assess the accuracy of HUMAnN's metabolic reconstructions, we constructed four synthetic metagenomic datasets with known functional profiles. Patterned on the study of Mavromatis et al [31], these included two low-complexity communities with 20 organisms each and two high-complexity with 100 organisms each. The former were manually chosen from representatives of the human microbiota, and the latter were randomly selected from KEGG high-quality bacterial genomes (see Methods, Supplemental Tables S2–3). Likewise, two of the communities contained organisms with equally distributed abundances, and two possessed lognormally distributed abundances to mimic physiological communities. A gold standard of functional modules and pathways as defined by KEGG [26] was assembled, comprising 13,980 gene families, 370 total small metabolic modules, and 309 large pathways. 251 and 303 of the latter, respectively, included at least four genes and were used here. Their presence in these communities was determined from KEGG genome annotations for the chosen organisms; these were used to evaluate a naive best-BLAST-hit metabolic reconstruction as compared with HUMAnN's inferences. In all cases, BLAST hits with >90% identity were discarded, preventing overconfident evaluations due to the gold standard's use of only well-characterized genomes and forcing a conservative estimate of HUMAnN's expected performance.

**Figure 2:**
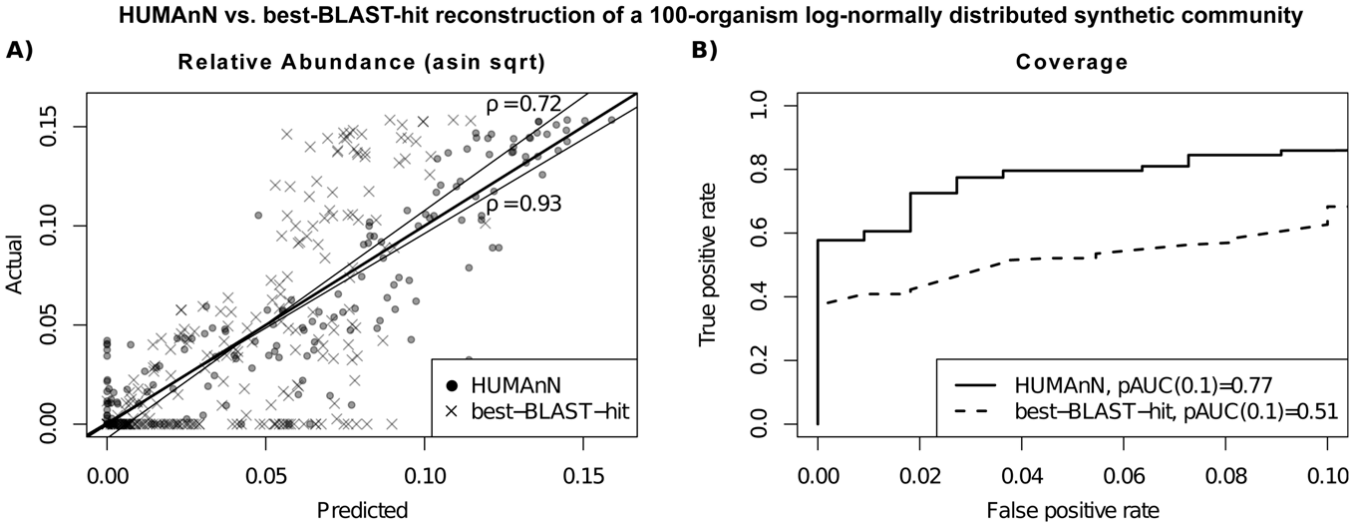
Accuracy of inferred module abundances and coverages using four synthetic metagenomes. An evaluation of HUMAnN's performance on a high-complexity mock community with a randomized log-normal distribution of 100 organisms as compared to an approach using the single best BLAST hit for each gene family and direct assignment to metabolic modules. Both A) correlation of inferred abundances (arcsine square root transformed for variance stabilization) and B) partial AUC at 0.1 false positive rate are high, outperforming single best BLAST hit functional reconstruction of microbial communities.

In all four communities, HUMAnN recovered metabolic module abundances with a correlation above 0.88 and a partial AUC at 10% false positives (pAUC10) above 0.73 (Figure 2, Supplemental Figure S2). Performance was generally comparable for large pathways (ave. ρ = 0.90 sd. 0.02, pAUC10 = 0.85 sd. 0.05), and in all cases HUMAnN outperformed reconstructions based on the best BLAST hit alone (Supplemental Figures S1–2). Although HUMAnN was not optimized to recover individual gene family abundances, it performed comparably to best-BLAST-hit at a correlation of 0.93 sd. 0.01 among the four communities. These synthetic communities were further used to refine the inclusion of computational steps within the HUMAnN pipeline and to assess the robustness of their parameter settings. For example, smoothing of low-abundance gene family frequency estimates proved to have surprisingly little overall impact, whereas MinPath was particularly critical for accurate pathway coverage determination (Supplemental Figure S1).

Within these four synthetic communities, only a few classes of gene families, modules, and pathways were recovered incompletely by HUMAnN. In the most complex staggered community, erroneous gene family calls included just 29 proteins missed as false negatives (of 5,640 total, 1.3% at abundance >10^−4^), typically short proteins <150AAs such as K13771 (the Rrf2 family transcriptional repressor) or K10533 (limonene-1,2-epoxide hydrolase). Likewise only 7 false positive proteins were detected based on closely related orthologs or strongly conserved domains (0.027% at >10^−4^), including K08721/OprJ (confused with K07796/cusC, blastp e = 3·10^−82^) and K11187/peroxiredoxin 5 (confused with peroxiredoxin 2, blastp e = 2·10^−19^). False positive modules (8, 3.3% at a 10^−4^ cutoff) were near-uniformly small gene sets overlapping with modules truly present (e.g. the urea cycle M00029, with five total genes and four present in the community). Conversely, false negatives (9, 3.7%) were most often small pathways present only in very low-abundance organisms, e.g. bicarbonate transport (M00321, four genes present at 0.022% relative abundance) or mannopine transport (M00301, four genes at 0.0014%). We specifically tuned HUMAnN to prefer false negatives to false positives based on these communities (see Supplemental Figure S2), and Figure 2 demonstrates the minimal impact of this choice even at low recall in the most complex community.

Fortunately, 89% of high-complexity (staggered) and 93% of low-complexity (even) modules were correctly called present or absent, and their inferred abundances were consistently well-correlated with the gold standard (Figure 2). These unsurprisingly included large, well-conserved pathways such as the ribosome (M00178) and polymerase (M00183), but also a variety of smaller specialized modules such as sugar transport (M00207, four genes, present in staggered/high-complexity at 0.25% and detected at 0.28%) and biotin biosynthesis (M00123, four genes, present at 1.7% and detected at 1.7%). Almost all large pathways were quantified with high accuracy, again due to their larger metagenomic footprint and detectability; examples included the TCA cycle (ko00020, 53 genes, present at 1.1% and detected at 1.2%) and base excision repair (ko00240, 152 genes, present at 1.1% and detected at 1.1%). In contrast, false positive rates for the best-BLAST-hit approach exceeded 23% of modules in the low-complexity community. Overall, as summarized in Figure 2 and in Supplemental Figures S1–3, this evaluation on synthetic metagenomes established that HUMAnN can accurately reconstruct community metabolic pathways and modules directly from short reads.

### Module-centric metabolic reconstruction of the human microbiome

We employed this optimized system to study microbial metabolism at seven body sites spanning the human microbiomes of 102 subjects. HUMAnN was applied to these data as described above, yielding the relative abundances and coverages both of functional modules and of full pathways, as well as the abundances of individual orthologous gene families. We first focused on analysis of small metabolic modules (ave. 11.2 sd. 9.2 genes), and 232 such modules were detected in at least one of the 649 samples; larger, more broadly defined pathways were also reconstructed from the HMP data and are described below. Both modules and pathways were reconstructed by HUMAnN as coverages (presence/absence calls on a zero-to-one scale) and as relative abundances for each sample (Supplemental Tables S4–5). The resulting metabolic reconstructions were complementary to the organismal compositions of the communities (see [24]) and provided a link between microbial environment and metagenomically prevalent pathways and metabolic potential.

**Figure 3:**
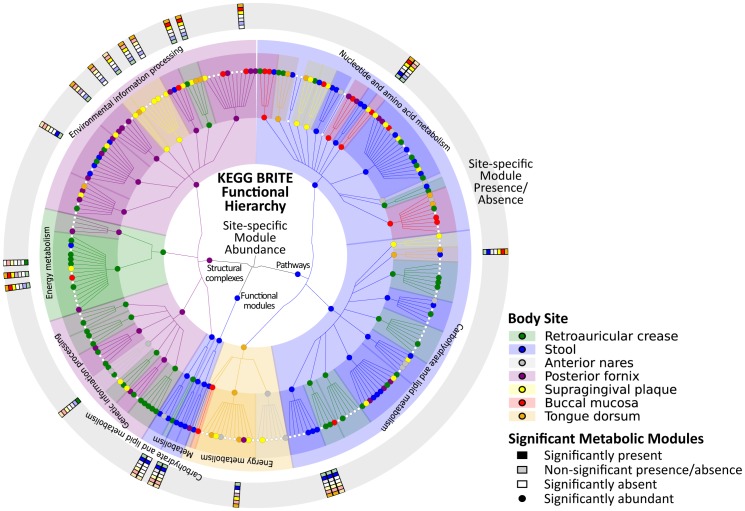
Metabolic modules differentially present or abundant in at least one body habitat of the human microbiome. Metabolic modules and pathways from the KEGG BRITE hierarchy [26] found to be differentially abundant (inner cladogram) or differentially covered (outer ring, presence/absence) in the human microbiome. The former were determined using LEfSe [23] and the latter by presence in at least 90% of samples with ≥0.9 coverage or absence in at least 90% with ≤0.1 coverage. Differentially abundant modules are colored by their most abundant body habitat. 168 significantly enriched module abundances were detected, in contrast to only 24 differentially covered.

In these data, we observed a core of 16 metabolic modules present at >90% coverage in >90% of samples (Supplemental Table S6), in contrast to essentially no specific microbes found to be core in this population [1]. However, in agreement with a gene family core from the gut microbiomes of an independent cohort [11], these modules comprise the functionality essential for microbial life: transcription (M00183, M00049-52), translation (M00178, M00360), transport (M00207, M00222, M00239), central carbon metabolism (M00001-2, M00006), and energy production (M00120, M00125, M00157, M00164). By relaxing the coverage threshold to 30% (expected to introduce few, if any, false positives; see 4.4), only 8 additional modules were included, demonstrating robustness to this threshold. Two of these extended the categories listed above; the remainder comprised sn-Glycerol 3-phosphate transport (M00198, a membrane lipid precursor [32]), the mannose and trehalose phosphotransferase systems (M00270 and M00276), spermidine/putrescine transport (M00299), early terpenoid biosynthesis (M00364), and threonine biosynthesis (M00018), the only amino acid module to meet this prevalence threshold. It should be noted that these 24 core modules are not the most abundant; for example, sn-Glycerol 3-phosphate transport reaches only a mean relative abundance of 0.001 sd. 0.002 across all samples, compared with the ribosome (M00178) at 0.03 sd. 0.008. Nor were they evenly abundant among habitats, as examples including phosphate and sugar transport (M00197 and M00222) are highly enriched in the posterior fornix as discussed below. The organisms performing the more specialized processes in this list also vary among habitats. Spermidine and putrescine are metabolized by the abundant *Streptococcus* spp. in the oral community, for example, processes that can play a role in halitosis [33]. However, this metabolic module is not present in reference genomes for the skin community's abundant *Corynebacterium* and *Propionibacterium* spp. [8], [26], and its abundance is instead correlated with that of the *Staphylococcus* reference genomes [24] (Spearman r = 0.87, n = 26, p = 1.8·10^−6^). Although this stringent core is itself moderately small, most other modules were consistently present or absent across body sites, with only 24 showing strongly differential coverage among habitats (Figure 3).

**Figure 4:**
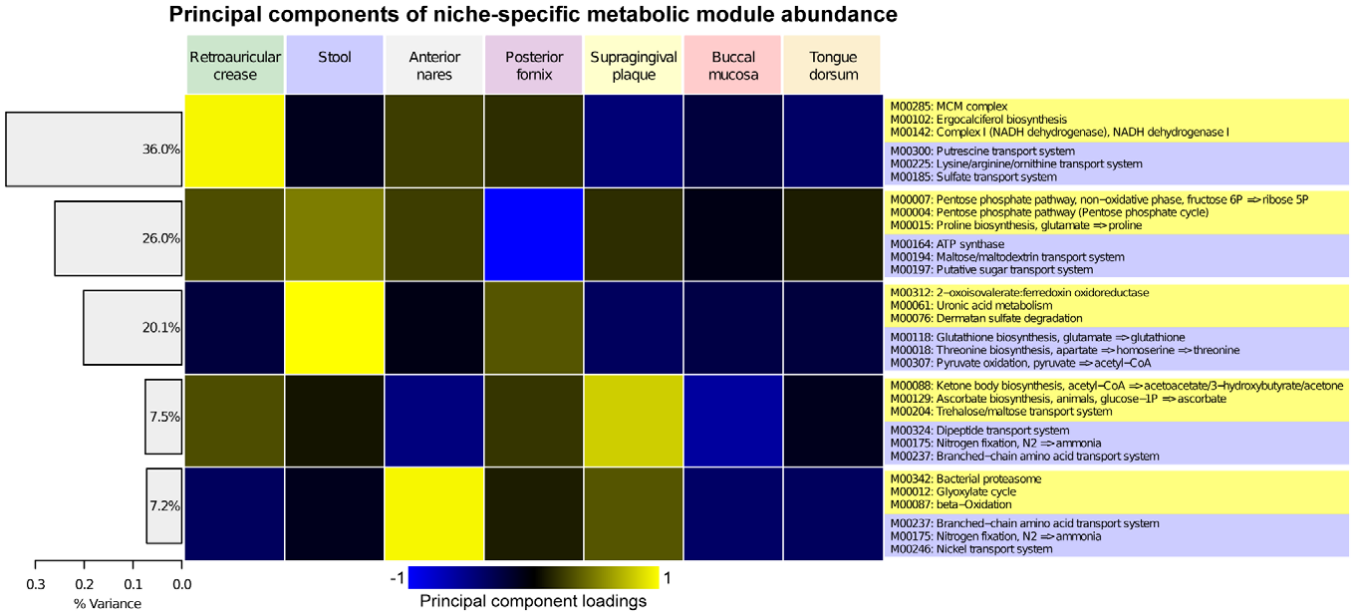
Patterns of abundance of functional modules in 649 metagenomic samples covering seven body habitats. A heatmap of the first five principal components (>95% variance) of module abundances averaged and normalized over each of the seven body sites. Cell color indicates positive (yellow) or negative (blue) variation, with the adjacent Scree plot showing the total variance in each component. The three most positively and negatively covarying modules contributing to each component are shown. Briefly, the first principal component differentiates the skin and gastrointestinal tract, the second differentiates the vaginal habitat, the third the gut, the fourth the supragingival plaque versus other oral sites, and the fifth the nares versus skin.

To investigate microbial functions over- or under-enriched within specific niches of the human microbiome, we determined modules differentially abundant in at least one body site using the LEfSe biomarker discovery suite [23] (Figure 3). Over two thirds (168, 67%) of detected metabolic modules varied significantly in abundance in at least one habitat, demonstrating the uniqueness of each body habitat's microbial environment (Supplemental Table S7). In addition to the detailed examples below, these included such diverse processes as arginine transport (M00229) and methionine biosynthesis (M00017) enriched in all three oral habitats, an enrichment for fungal transcription (M00181) and translation (M00177) in the skin and airways, and a strong depletion of pyruvate (M00307) and second carbon oxidation (M00011) in the anaerobic gut and vaginal sites. Several overall patterns of co-variation are shown in Figure 4, where the first principal component captured primarily eukaryotic modules found only on the skin and often nares (including, intriguingly, vitamin D biosynthesis, M00102). Interestingly, it also included metabolism abundant throughout the digestive tract (i.e. oropharynx and gut), such as putrescine (M00300) and sulfate transport (M00185) [23]. The second principal component described functionality depleted in the low-complexity vaginal habitat, and the third comprised processes enriched in the gut, both discussed below. The three oral habitats are often functionally similar (apparent in components 1–3), and the fourth emphasized modules unique to the tooth surface, the only microbially colonized hard surface assayed here, in contrast to the mucosal and tongue soft tissues [23]. While the skin and nares were likewise often similar, the final principal component shown in Figure 4 differentiates the two. These summaries show that while a wide range of microbial metabolism is present throughout the human microbiome, specific subsets of this functionality are selected for by the unique combinations of nutrients, immune pressures, and environmental exposures present at each body site.

Ecological and phylometagenomic studies of organismal abundance often employ summary statistics including species richness, evenness, or diversity to characterize and compare communities [34]. These quantify how many distinct types of organisms occupy a community, the uniformity of their relative abundances, or both, respectively. These measures were historically adopted from macroecology into microbial ecology, and while the former has included assessments of functional diversity [35], [36], it has been proposed for microbial communities [37], [38] but not yet widely adopted in metagenomic studies. Functional diversity measures are thus calculated by HUMAnN as a novel means of profiling microbial community structure, with some differences from their applications in macroecology as described in the Discussion. A simple measure of richness is calculated by summing module coverage scores within each sample [34], and Pielou's evenness [39] and the Shannon and inverse Simpson [34] diversity measures are calculated from module abundances. As observed qualitatively by Turnbaugh et al [5] and analyzed quantitatively by the HMP [1], microbial metabolic function differs significantly less among subjects than does organismal diversity within each habitat. Ecological summary statistics of community function may thus represent a unique descriptor of metagenomic data complementary to standard organismal diversity measures.

### Association of community function with microbial environment and host phenotype: glycosaminoglycan degradation is uniquely abundant in the gut, and multiple pathways correlate strongly with vaginal pH

Modules performing glycosaminoglycan (GAG) degradation in the gut were among those most differentially abundant among habitats. These included chondroitin sulfate degradation, dermatan sulfate degradation, and keratan sulfate degradation, as well as the related uronic acid metabolism. All four of these modules are involved in animal proteoglycan degradation for microbial carbohydrate utilization [40], and they were present in high abundance in the gut (coverage >0.9 in 136 stool samples, 100%) and rare or completely absent in other body sites (coverage <0.1 in 131 samples, 26%; abundance <0.001 in 512 samples, 99%). This degree of specificity was unusual in the HMP dataset - as mentioned above, relatively few modules were present or absent in only one such body site. However, it provides a readily identifiable example of community metabolism associated with a specific clade, as glycosaminoglycan degradation is known to be enriched among the *Bacteroides* species [5], [41]. The model *B. thetaiotaomicron* alone, for example, carries nearly 90 different polysaccharide utilization loci, many targeting dietary starches, but at least 16 specific to host mucin O-glycan degradation [42]. As the *Bacteroides* are one of the predominant genera in the gut microbiome [43], are largely characteristic of only that body site, and are abundant in the HMP gut samples [1], they are highly likely to be responsible for this niche-specific metabolism.

**Figure 5:**
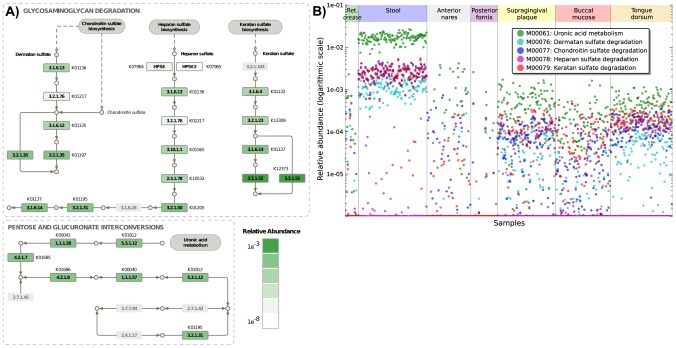
Gene- and module-specific reconstruction of glycosaminoglycan degradation specific to the gut microbiota. A) Individual gene family abundances for four gut-specific high abundance modules: chondroitin, dermatan, and keratan sulfate degradation (glycosaminoglycan degradation, also including heparan sulfate), and uronic acid metabolism (occurring directly downstream in the pentose and glucuronate interconversion pathway). Relative abundance is shown from dark (high) to light (low) green, averaged over 136 stool microbiomes, with enzymes not present in the KEGG Orthology in gray. Heparan degradation is absent specifically due to the lack of heparanase (K07964-5), but no one gene family is otherwise responsible for the high abundances of the remaining four modules in the gut, despite several shared enzymes (e.g. beta-glucuronidase, K01195). B) Relative abundances of all five modules in all body habitats and samples, demonstrating gut-specific prevalence. Despite the close connections among these pathways, they show distinct patterns of relative abundance specific to the gut and covary at very low abundance in the oropharynx.

HUMAnN's reconstruction further allowed examination of the individual orthologous gene families participating in these interrelated pathways. Chondroitin and dermatan sulfate degradation share the greatest overlap with 3 KO families, including beta-glucuronidase (K01195). Beta-glucuronidase is prevalent in the gut, averaging 0.03% sd. 0.01% in our data, in contrast to all other nonzero KOs (ave. 0.007% sd. 0.03%). It is one of the primary enzymes involved in metabolism both of food matter and of pharmaceuticals [44], as well as mediating effects ranging from dietary cancer risk [45] to antibiotic activity [46]. This enzyme also links uronic acid metabolism with the rest of pentose and glucuronate processing, the former being the most abundant module of these gut-specific examples. Uronic acid, like the GAG sulfates, is a component of dietary fiber and glycoproteins degraded by intestinal bacteria [42]. In contrast, heparan sulfate degradation, an additional module included in glycoprotein degradation as defined by KEGG, is present at only low abundance among all body sites, in spite of sharing nearly half of its enzymes with the other three modules (ave. 7·10^−4^% sd. 0.003% in the gut, <10^−5^% elsewhere). This is due exclusively to the absence of the module's input enzyme, heparanase (K07964-5), a typically eukaryotic gene family implicated in tumor metastasis [47]; the healthy commensal microbiota may thus lack this activity in order to avoid undesirable inflammatory and immune response in the gut [48]. Conversely, the abundances of the four gut-specific GAG degration modules were not driven by one specific gene family (Figure 5), ranging from the most abundant beta-hexosaminidase (K12373, ave. 0.3% sd. 0.1%) to the low-abundance outlier L-iduronidase (K01217, ave. 2·10^−5^% sd. 3·10^−7^%). Given this ubiquity, it should be noted that GAGs possess wide-ranging activities including anti-cancer [49] and antimicrobial [50] properties; the abundance of degrading enzymes in the gut of any particular individual thus has the potential to affect the efficacy of GAG drugs [41], [51]. The specificity and prevalence of these four modules in the gut microbiota provides one example of HUMAnN's ability to identify community metabolism across hundreds of samples and link it to individual microbial gene families.

We next examined modules differentially abundant in the 53 HMP vaginal posterior fornix samples (Supplemental Table S7). Enriched functions included phosphate, glutamate/aspartate, and phosphotransferase transport systems (M00222, M00230, M00276, M00277, M00287); other functions such as arginine and amino acid transport (M00229, M00237) were significantly depleted. The posterior fornix community's metabolic pattern was perhaps the most distinct of the habitats examined here, in agreement with the high degree of biochemical specialization (and thus intercellular transport) observed in the low-diversity vaginal community [7]. Perhaps most interestingly, the misleadingly labeled “nitrogen fixation” module (M00175) was overabundant in the posterior fornix, as well as in buccal mucosa and stool. This module can be driven by any one of four gene families: K00531, K02588, K00536, or the complex K02586+K02591. The low number of genes involved would render its detection prone to noise; however, as described below, this module is detected very consistently only in women with vaginal pH≥4.0, and then only due to either K00536 (Enzyme Class EC 1.19.6.1, nitrogenase - flavodoxin) or, in a minority of cases, K02588 (EC 1.18.6.1, nitrogenase). These two gene families were mutually exclusive when detected, and the most common family K00536 is only included in the current reference genomes for *Bacillus cereus*, *B. thuringiensis*, and the archaeon *Archaeoglobus fulgidus*, none of which were detected in any vaginal samples [9]. While it seems unlikely that these enzymes are contributing to canonical nitrogen fixation per se, flavodoxin has been observed to be involved in the catalysis of nitric oxide production in the gut microbiota [52], [53], and disrupted amine production in conjunction with elevated vaginal pH is a standard symptom of bacterial vaginosis [7]. In combination with the vaginal pH-associated modules below, this provides one example of microbial functionality linked to host phenotype that could not be recovered based on reference genomes, community structure, or phylometagenomic assays alone.

Given their lower diversity, the functional profiles of these vaginal microbiomes proved to be particularly informative when associated with host phenotype and microbial membership. Taxonomic profiles detailing the abundances of microorganisms in these communities were available from the HMP's 16S rRNA gene surveys [9], from mapping of metagenomic sequences to reference genomes [24], and from previous studies of the vaginal flora [7]. Specifically, Ravel et al [7] showed the presence of five microbiome types among the vaginal communities of reproductive age women, with four clusters dominated by different *Lactobacillus* species and one with low levels of all lactobacilli. Communities dominated by *L. crispatus* corresponded to lower pH (<4), which was also the case in the HMP posterior fornix data [1]. We observed a very tight co-clustering of the metabolic repertoires of these communities with the abundances of these five groups' characteristic species (Supplemental Figure S5), suggesting that in this specialized microbial niche, there is a particularly close association of community structure and function. To expand on this, we performed a comprehensive test of all 232 modules against the HMP's clinical metadata (phs000228.v3.p1 [54]) to uncover associations between microbial metabolic profiles and host phenotype. Available metadata included gender, age, BMI, geographical location, and others, as well as the pH of the posterior fornix and vaginal introitus in women. The latter were again strongly linked not only to community structure, but also with the abundances of several metabolic modules. Specifically, pH correlated with the abundances of N-acetylgalactosamine II phosphotransferase (M00277), proline biosynthesis (M00015), and again “nitrogen fixation” (M00175), while phosphate transport (M00222), peptide/nickel transport (M00239), and lysine biosynthesis (M00016) were associated with lower pH. Species within the *Lactobacilli* are known to specialize in exactly these areas of nutrient uptake and carbohydrate metabolism [55], [56], and many of these differences can be observed directly within finished genomes (e.g. lysine biosynthesis is present in *L. crispatus* and lacking in *L. gasseri* [26]). It is currently near-impossible to obtain complete genomes for all organisms in complex communities, however, again emphasizing the utility of metagenomic functional reconstruction for direct association of community function with habitat and host phenotype.

A surprising feature of these data, however, was the observation of few other robust correlations between microbial metabolic abundances and host phenotype outside of the vaginal community, in contrast to recent studies of the gut microbiota [12] (Supplemental Table S1). Of all phenotypic associations with metabolic modules tested in the HMP stool microbiomes, only two reached significance (Spearman FDR q<0.1), links between diastolic blood pressure and nickel transport (M00246) and between pulse rate and methionine degradation (M00035). Although these are within the range of false positives expected at this level of stringency, recent observations of the gut microbiota in atherosclerosis [57] have also detected borderline significant shifts in organismal composition. Among other body sites, the only additional associations were correlations with BMI among the skin community: lysine biosynthesis (M00016) and sulfate transport (M00185). Although the retroauricular crease represents our smallest sample size (n = 26), these were significantly stronger (p<10^−4^) than any metabolic association with BMI in the stool community (n = 126, lowest p<10^−3^), with the metagenomic abundance of lysine biosynthesis consistently increasing and that of sulfate transport decreasing at greater BMI. Additional associations beyond this false discovery rate are included in Supplemental Table S1 and include several modules detected only at higher read counts, emphasizing the need for sufficient sequencing coverage when rare metabolic functions (as opposed to abundant taxa) are of interest. As has been shown for genotype [58] and gene expression [59] data, phenotype can be difficult to reproducibly associate with high-dimensional genomic or metagenomic features, particularly in large cohorts with complex population structure or when using multivariate models [60]. It is also critical to note that the HMP population was strictly screened for disease-free individuals [9], increasing phenotypic homogeneity and precluding the detection of microbial function perturbed in dysbioses. Further studies targeted to specific phenotypes and microbial communities of interest will be necessary to better understand the relationship between human microbiome membership, metabolism, and host phenotype.

### Relating larger metabolic pathways to functional modules and gene families: implementation of metabolic processes varies by niche

The analyses described up to this point, and the primary outputs of HUMAnN for metagenomic samples, focus on the coverages and abundances of small functional modules and individual gene families. Such modules are typically defined to carry out a specific metabolic step, such as production of a single amino acid from its immediate precursor. They contain an average of only ∼10 genes and are structured to include both “and” relationships (multiple gene products that must function together in a complex or sequential pathway) and “or” relationships (alternative enzymes that can catalyze the same reaction). However, KEGG and other functional catalogs also define large pathways with up to several hundred genes that lack the complex combinations of relationships used to define small modules. HUMAnN can additionally recover coverages and abundances for any such pathways represented by unstructured sets of gene family identifiers. To this end, we analyzed 297 KEGG pathways present in the seven HMP body sites, containing 52 sd. 48 genes on average. While HUMAnN successfully recovered coverage and abundance information for these larger pathways in the HMP data (Supplemental Tables S8–9), we often found them to be too broadly defined for adequate analysis of mixed microbial communities, arguing for a focus on smaller functional modules, biosynthetic clusters [61], and orthologous gene families.

Demonstrating these larger pathways' lack of specificity, 190 (64%) were at most 50% covered among all 649 samples; that is, although portions of the pathways were detected, at least half of the associated gene families were missing in every assayed microbial community. Of the remaining 107 pathways, 96 (90%) had a coefficient of variation below one, indicating variation in coverage lower than their mean across all samples. In other words, only small portions of most KEGG pathways are present in the human microbiome. Those pathways that were consistently present tended to be so broadly defined (e.g. ko02060, phosphotransferases, or ko00030, the entirety of pentose phosphate) that they provided too coarse a view with which to observe metabolic variation and niche specialization among body sites. Nevertheless, applying the same significance criteria as described above, 196 pathways (66%) were differentially abundant in at least one body site using LEfSe. These are detailed in Supplemental Table S7 and are for the most part analogous to the more specific small modules described above.

In contrast to this variation, however, we were again able to recover a subset of “core” pathways with moderate coverage and low variability across all sites of the human microbiome. The pathways with the lowest coefficient of variation across all samples in our data represented surprisingly diverse biochemistry, including terpenoid biosynthesis (ko00900), RNA degradation (ko03018), pyruvate metabolism (ko00620), and one-carbon metabolism (ko00670). These pathways were all present at an average of at least 0.5% relative abundance over all samples, with average coverages from 41% (of 75 genes in ko03018) to 77% (of 73 genes in ko00620). Each of these pathways followed a very characteristic pattern: their overall pathway abundance remained near-constant across body site niches, while the modules implementing each pathway varied significantly. Terpenoid biosynthesis, for example, can be performed either by the mevalonate (module M00095) or by the non-mevalonate (M00096) module; both were present in the oropharynx and skin, only the former was present in the posterior fornix, and only the latter in the gut (Supplemental Table S5). Likewise, pyruvate metabolism includes portions of the citric acid cycle (M00173), absent from the posterior fornix and rare in the gut and skin; pyruvate oxidation (M00307), absent from the posterior fornix and rare in the gut; and pyruvate ferredoxin oxidoreductase (M00310), present only in the nares and gut. This trend complements the relationship between organismal and functional diversity described above and by the HMP overall [1], in which the communities at each body site of the human microbiome vary extensively, but the pathways needed for microbial life within these niches remain relatively stable. However, this result shows that the specific metabolic modules implementing stable, broad pathways tend to be specialized within each body site's microbial environment.

This pattern extended to individual gene families as well; while the pathways above demonstrated low variability across all body sites, other pathways were enriched at specific sites but possessed low inter-subject variability in the HMP population (Supplemental Table S9). These include, for example, glutamate metabolism in the gut (ko00250), which has been previously observed to be enriched in the stool microbiota [62]. Here, it showed low inter-individual variability in the gut, although as above, its product glutamate serves as input for several modules that were themselves highly variable among subjects. Among others, these included proline biosynthesis (M00015); glutathione biosynthesis (M00118); glutathione transport (M00348); and portions of the TCA cycle (M00009–M00011 and M00311). We determined individual carbohydrate active enzyme (CAZy) gene families correlated with these modules by comparing them with CAZy abundances derived independently from the HMP metagenomic data [21]. Intriguingly, each of these modules in the gut correlated significantly (Spearman FDR q<0.05) with multiple CAZy families, almost none of which included enzymes within the modules themselves (Supplemental Table S10). In particular, six CAZy-module pairs had significant positive or negative correlations in all seven body sites: proline biosynthesis with GH5 (cellulase) and GH18 (chitinase); 2-oxoglutarate ferredoxin oxidoreductase with GH28 (galacturonases) and GH97 (α-glucosidase and α-galactosidase); and glutathione transport with GH3 (β-glucosidase) and GH18.

Summarizing these three results, glutamate metabolism falls within a class of 57 pathways present in multiple niches within the human microbiomes sampled here but enriched in specific environments (e.g. the gut). 101 total large pathways varied little in site-specific abundance among individuals, but smaller modules within them (such as proline biosynthesis) showed greater inter-subject variability. Finally, these module-specific changes in abundance almost always (99% of all modules) correlated with one or more individual CAZy gene families detected within the same metagenome independently of the pathway's constituent genes. Together, these data suggest that while the basics of microbial metabolism remain stable among human microbiome body sites and individuals, the modules and enzymes operating as specific metabolic producers and consumers within pathways vary among environments and subjects to adapt to changing nutrient and metabolite availabilities [10].

## Discussion

Culture-independent metagenomic sequencing of microbial communities provides a wealth of data regarding their potential biological functions, particularly as studied for the human body by the Human Microbiome Project. Here, we have described the development of the HUMAnN methodology for high-throughput metagenomic functional reconstruction and its application to 649 communities from 7 body habitats sequenced as part of the HMP. Validation of HUMAnN's accuracy using four additional synthetic communities of increasing complexity demonstrated its ability to quantify both pathway presence and relative abundance, with correlations to true abundances >0.9. When analyzing the human microbiome, relatively few modules were specifically present or absent in any one body habitat, but over two thirds varied in abundance by habitat and 24 were core to all hosts and habitats. Less variation was evident among hosts, although we demonstrated one example in which nutrient transport and mechanisms of central carbon metabolism were strongly associated with vaginal pH. HUMAnN's functional reconstructions include the abundances of large, general pathways, smaller and more specific metabolic modules, and individual orthologous gene families; each data type proved to show distinct patterns of variation among body sites and to provide a different perspective on underlying microbial community function.

Characterization of microbial communities by large-scale shotgun metagenomic sequencing is a relatively recent advance, and computational methods for assessing these data in terms of biological function are under active development. Several previous studies have analyzed individual reads or assembled contigs using direct annotation by BLAST to orthologous gene families [5], [11] or to proxy genes [63]. Other computational pipelines such as MG-RAST [14] and MEGAN [15] do include full pathway reconstruction, generally using approaches that rely on the single best BLAST hit for each metagenomic read. Here, HUMAnN scaled easily to provide module and pathway reconstructions for >3.5 Tbp of HMP metagenomic data by avoiding metagenomic assembly and employing an accelerated translated BLAST implementation, requiring a total of approximately 13,000 CPU-hours for sequence search and 175 for metabolic reconstruction (750 and 55K sequences/second, respectively). HUMAnN is not dependent on any particular BLAST implementation, however, and provides default support for NCBI BLAST, MBLASTX as employed here, MAPX (Real Time Genomics, San Francisco, CA), and USEARCH [27]. In each case, the approximations used to accelerate search against a functionally characterized orthologous sequence database are mitigated by considering all read-to-sequence hits in a weighted manner. This leaves overall gene family abundance recovery essentially unchanged while improving full module/pathway recovery, as ambiguous BLAST hits can be resolved later in the reconstruction process when more information is available (Supplemental Figure S2). Further, each of HUMAnN's processing modules incorporates one or more types of additional knowledge, e.g. pathway parsimony by means of MinPath [22] and subsequent automatic taxonomic limitation based on BLAST organismal abundance profiles. These steps are not guaranteed to be optimal in all situations - taxonomic limitation, for example, might degrade performance in environments rich in novel or rapidly evolving organisms - but they are heuristics designed to improve reconstruction in most cases. They thus generally take advantage of the compositional information that can be leveraged when combining multiple gene family sequences into a single module or pathway, decreasing the noise potentially arising from examining single best BLAST hits.

It should be noted that when analyzing metagenomic data as described here, HUMAnN reconstructs a profile of a microbial community's metabolic potential, not its metabolic activity per se. The abundances of gene families and pathways inferred by the system describe only the enzymes encoded by one or more microbial genomes, and their relationship to realized transcriptional or protein activity may not be straightforward in the absence of additional metatranscriptomic, metaproteomic, or metametabolomic data [64]. However, these metagenomic gene family and module abundances are appropriate as inputs into more sophisticated metabolic network and systems biology models, which have recently begun to incorporate features such as predicted compartmentalization, small molecule transport, and multi-organism interactions in microbial communities [65], [66]. HUMAnN as described here was designed to infer a permissive superset of community function that does not yet include realized transcriptional activity or organismal compartmentalization, and we hope to incorporate these features during future work. It should be emphasized that HUMAnN as currently implemented is appropriate for analysis of metatranscriptomic data from short sequence reads as well, from which it will reconstruct the abundance of actively transcribed gene families or pathways within a microbiome.

The results produced by HUMAnN and analyzed above for the human microbiome include the application of several community diversity measures to microbial function. Such measures are typically applied instead to organismal abundances, where α-diversity summarizes complexity and types of different organisms within a community and β-diversity the similarities (or differences) between multiple communities' structures [34]. Such organismal diversity measures have been very successful in describing properties of the human microbiome in large populations, such as the greater similarity of children's and parents' microbiomes [5] or reduced microbial diversity in conditions such as Crohn's disease [67]. Conversely, ecological functional diversity has been developed primarily in macroecology, specifically as applied to phenotypic traits [35], [68]. To our knowledge, however, this represents the first application of α-diversity measures to molecular function within microbial communities and specifically to the human microbiome. The HMP consortium has contrasted the functional diversities reported above with comparable organismal diversity measures at the genus, species, and strain levels throughout the human microbiome [1]. Their results suggest that functional diversity is lower than phylogenetic diversity both within and between communities throughout the human microbiome; that is, the microbes within this human population vary more than do the biological processes carried by their metagenomes. It must be noted that this conclusion speaks so far only to the disease-free HMP population, however, and only to the subset of characterized orthologous gene families currently analyzable by HUMAnN. Further variability in the functional potential of the microbiota may certainly remain to be found in its substantial carriage of uncharacterized gene families (estimated as high as 80% [11]) and during disruptions of host health.

A key consideration during our development of the HUMAnN pipeline was versatility; the software implementation can easily be extended to assess any functional catalog, characterized sequences, or metagenomic sequences (e.g. 454 reads). In other analyses by the HMP, additional protein databases including MetaCyc [28], CAZy [21], virulence related proteins [69], and antibiotic resistance genes [70] were all processed using HUMAnN. MetaCyc, for example, includes both characterized sequences and metabolic modules, for which HUMAnN reconstructed coverages and abundances; other databases included no explicit pathway groupings, and gene families were used directly to examine differential abundance. While these smaller databases are less appropriate for quantitative evaluations or broad metabolic reconstruction, they can be used with HUMAnN to provide focused coverage of specific biological areas. As detailed above, in addition to its primary abundance and coverage outputs, HUMAnN by default calculates a number of basic ecological summary statistics as applied to community functional profiles; it also produces detailed gene-level outputs for each community that can be directly imported into the JCVI Metagenomics Reports (METAREP) [71] software. All components in the pipeline, including taxonomic limitation, are entirely data-driven; the methodology can therefore be used for functional reconstruction on any genomic data from microbial or eukaryotic organisms, although in a single-organism setting, there are not clear benefits over standard genome annotation pipelines. However, individual modules (such as gap filling or the inclusion of multiple BLAST hits) can be manually activated or deactivated by the user for particular datasets. Importantly for microbial communities, HUMAnN can also be used on other data types, including metaproteomic or metatranscriptomic sequences; we anticipate HUMAnN being useful in the reconstruction of pathway activities in transcriptomic sequences from different environmental communities, for example.

In closing, we would like to emphasize that HUMAnN's current approach to microbial community functional reconstruction is explicitly independent of the organismal membership of these communities. It was designed to complement taxonomic classifications of community structure, and integration of community function with membership is an area of further ongoing work [15]. Particularly in the human microbiome, full genome sequences are available for many reference strains isolated from multiple body sites, which has already allowed community membership to be analyzed simultaneously in metagenomic and 16S taxonomic marker sequences [1], [24]. By combining membership with functional reconstruction, specialized processes in specific habitats or hosts, for example, can be correlated with the organisms providing or dependent on these aspects of community function. While the general applicability of Beijerinck's 1913 hypothesis [72] that, “Everything is everywhere, and the environment selects,” is still unclear, we speculate that it may prove to be more broadly accurate for microbial function than for microbial organisms. That is, there may be a moderately stable pool of core microbial pathways, present in all communities but implemented by different organisms and gene families, with relative abundance (and activity) determined by the local selective pressures of each microbial habitat. This appears to be at least somewhat the case in the human microbiome, and further investigation will determine whether this pattern holds for the functional profiles of broader classes of microbial communities.

## Supporting Information

Figure S1 **Evaluation of parameter settings and processing modules in the HUMAnN pipeline.** Four synthetic metagenomes (high and low complexity, equally and lognormally distributed organismal abundances) were used as a gold standard to evaluate variants of the HUMAnN pipeline for both metabolic modules (Mod.) and full KEGG pathways (Path). The accuracies of relative abundance estimates (left) were evaluated using Pearson correlation with the gold standards (Supplemental Table S3), whereas the coverage (right) was evaluated using partial AUC at 10% false positives in order to specifically prevent erroneously high-confidence false positives. The choices evaluated here include whether to assign genes to modules/pathways using MinPath (MP) or naively, inclusion of taxonomic limitation (Tax) without or with gene copy number correction (TaxC), add-one (Sm) or Witten-Bell (SmWB) abundance smoothing, and biological gap filling using pathway medians (GF) or averages (GFAve). The final HUMAnN pipeline that achieved the best overall performance is highlighted in bold and consists in the sequential execution of MinPath, taxonomic limitation with copy number correction, and median-based gap filling.
(PDF)

Figure S2 **Performance of HUMAnN abundance and coverage inference as compared to a best-BLAST-hit (BBH) approach.** The HUMAnN pipeline for KEGG metabolic modules and pathways was compared to a best-BLAST-hit approach to module reconstruction. Evaluations of abundance (left columns, by Pearson correlation) and coverage (right columns, by partial AUC at 10% false positives) are summarized in the first row. Subsequent rows show full scatterplots with regressions (left, abundances) and ROC curves (right, coverages) for each individual method over the entire gold synthetic community gold standards. These include A) high-complexity (100 organisms) staggered abundances B) low-complexity (20 organisms) staggered, C) high-complexity even abundances, and D) low-complexity even.
(PNG)

Figure S3 **Accuracy of HUMAnN relative abundance predictions for individual KOs.** Predicted versus actual abundances sunflower plotted for each of the 13,980 KEGG Orthology gene families (KOs) evaluating four E-value weighting schemes (HUMAnN's default p-value, inverse E-value, bitscore, and sigmoid E-value) and best-BLAST-hit alone in the A) the staggered high-complexity synthetic community, B) the staggered low-complexity, C) even high-complexity, and D) even low-complexity. Pearson correlation coefficients and regressions of arcsine square root transformed values are also reported. The performance of HUMAnN on the overall population of individual genes did not differ significantly from that of a best-BLAST-hit approach, although the preservation of additional information from multiple hits for later use (using any weighting scheme) does improve module and pathway abundance recovery (Supplemental Figure S2).
(PNG)

Figure S4 **Evaluation of individual HUMAnN processing modules on 10 synthetic metagenomes.** An additional 10 synthetic metagenomes were generated with high-complexity (100 organisms) and random lognormally distributed abundances. These were searched against KEGG protein sequences using USEARCH, allowing multiple hits with maximum e-value 1. All combinations of select HUMAnN modules were then assessed, including best-BLAST-hit versus multiple hits weighted by p-value and the presence or absence of taxonomic limitation with or without copy number normalization. HUMAnN default settings are highlighted in gray. Processing steps recapitulated their behavior as observed in Supplemental Figure S1.
(PDF)

Figure S5 **Co-clustering of metabolic pathways and microbes from community-specific genera in the vaginal microbiome.** Hierarchical clustering (average linkage using uncentered Pearson correlation) was applied jointly to the eight signature organisms from the five lineages known to characterize different microbiome types [7] and to the 62 metabolic modules correlated with these taxa in the posterior fornix microbiome. All relative abundances are row z-score normalized for visualization. Differentially abundant metabolism varies directly in proportion to these signature taxa, which themselves group the 52 samples into five distinct microbiome types. These are in turn correlated with the host vaginal pH phenotype, suggesting the specialization of these organisms with respect to metabolic function in the maintenance of community structure and environmental pH.
(PNG)

Table S1 **Significant associations between microbial function and host phenotype in seven habitats of the human microbiome.** Each row reports a significant body-site-specific association between microbial functional module abundance and host metadata, including biometrics (gender, BMI, etc.) as well as sample processing (e.g. sequence depth). P-values were calculated using Spearman's r for continuous metadata and the Kruskal-Wallis nonparametric ANOVA for categorical features as reported in the Methods, with Q-values representing a Benjamini-Hochberg false discovery rate correction for multiple hypothesis tests.
(XLSX)

Table S2 **Community composition of four synthetic metagenomes used to evaluate performance of metabolic reconstruction.** Microbes for the low-complexity (20 organism) community weree hand-chosen from clades representative of the human microbiome. Their relative abundances in the staggered community were likewise chosen to be roughly representative of physiological occurrence rates. Organisms in the high-complexity (100 organism) synthetic community were randomly selected from KEGG high-quality manually curated finished genomes. Their abundances in the staggered community were randomly generated from a lognormal distribution. These synthetic community designs for computational performance evaluation were inspired by those of [31] as adapted to the complexity and phylogeny of the human microbiome.
(XLSX)

Table S3 **Gold standard abundances of KEGG gene families, pathways, and modules for four synthetic metagenomes.** A ground truth of genes, small functional modules, and large pathways in each of four synthetic metagenomes was calculated from the organismal compositions in Supplemental Table S2 as follows. The abundances of gene families were computed by multiplying the frequency of each KEGG Orthology gene family in organisms' reference genomes by the organism's relative abundance (even or staggered) in each community. Module presence/absence and abundance was determined based on conjunctive normal form satisfaction of constituent gene presence/absence and relative copy number, respectively. Pathway presence/absence was used as defined by KEGG, with abundance as multiplied by each organism's relative abundance within the communities.
(XLSX)

Table S4 **HUMAnN coverage estimates for functional modules in the human microbiome.** Inferred module presence/absence confidence values for each of 227 KEGG modules in 649 samples spanning seven body sites across the human microbiome.
(XLSX)

Table S5 **HUMAnN relative abundance estimates for functional modules in the human microbiome.** Inferred module abundance values for each of 250 KEGG modules in 649 samples spanning seven body sites across the human microbiome.
(XLSX)

Table S6 **Core and differentially covered modules in the human microbiome.** Metabolic modules determined to be core and variable across body sites based on coverage estimates from the HUMAnN pipeline. Core modules were defined with coverage ≥0.9 (or at a lower stringency ≥0.3 threshold) in ≥90% of the samples from each body site. Variable modules were defined to be present (as above) in at least one body site and absent (≤0.1 in ≥90% of samples) in at least one body site. 24 samples. Using these definitions, 16 modules were core across all body sites at coverage 0.9 and 24 at 0.3, and 24 modules were variably covered among body sites.
(XLSX)

Table S7 **Modules and pathways differentially abundant in at least one body site as determined by LEfSe.** We applied the LEfSe [23] biomarker discovery tool separately to functional module and pathway abundances from HUMAnN to determine those over- or under-enriched in at least one body site. Default statistical parameters of α = 0.05 and LDA score 2.0 were used. This table reports features found to be differential in at least one body site and the site at which they were most abundant.
(XLSX)

Table S8 **HUMAnN coverage estimates for metabolic pathways in the human microbiome.** Inferred pathway presence/absence confidence values for each of 281 KEGG pathways in 649 samples spanning seven body sites across the human microbiome.
(XLSX)

Table S9 **HUMAnN relative abundance estimates for metabolic pathways in the human microbiome.** Inferred pathway abundance values for each of 297 KEGG pathways in 649 samples spanning seven body sites across the human microbiome.
(XLSX)

Table S10 **Significant associations between KEGG modules and individual carbohydrate active enzyme (CAZy) gene families.** Statistically significant Spearman correlations between module abundances (from HUMAnN) and CAZy abundances (independently generated from the same metagenomic data by [21]) were identified across all body sites (FDR q<0.05). Note that many metabolic functions within every body site correlate strongly with CAZy abundances, even for the many pathways that do not themselves include the correlated CAZys.
(XLSX)
