# Supplementary figures and images for: Metabolic Reconstruction for Metagenomic Data and Its Application to the Human Microbiome
Source: PLoS Comput Biol. 2012 Jun 13;8(6):e1002358. doi: 10.1371/journal.pcbi.1002358 (PMC3374609; doi:10.1371/journal.pcbi.1002358)

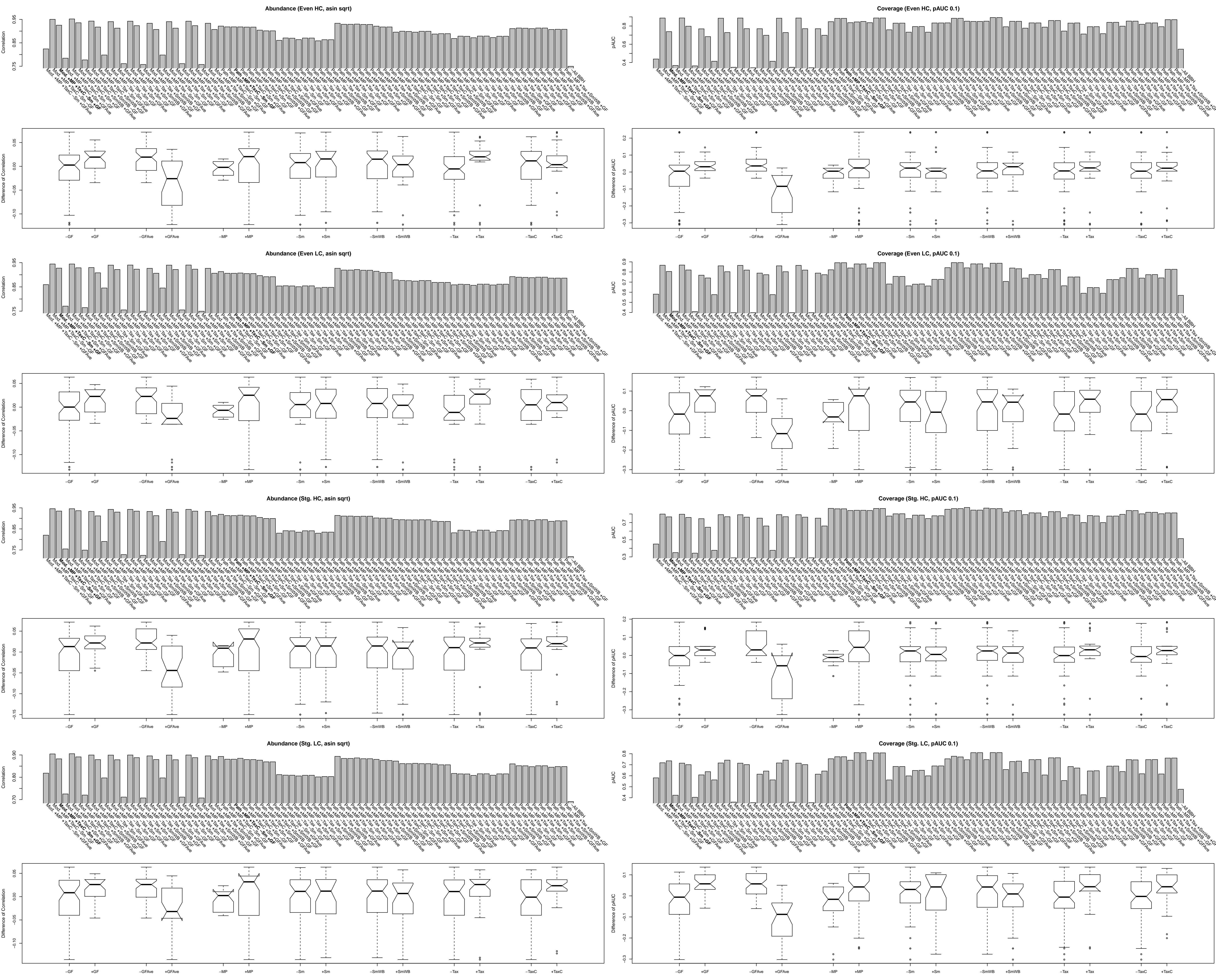

Supplement: Figure S1 — Evaluation of parameter settings and processing modules in the HUMAnN pipeline. Four synthetic metagenomes (high and low complexity, equally and lognormally distributed organismal abundances) were used as a gold standard to evaluate variants of the HUMAnN pipeline for both metabolic modules (Mod.) and full KEGG pathways (Path). The accuracies of relative abundance estimates (left) were evaluated using Pearson correlation with the gold standards (Supplemental Table S3), whereas the coverage (right) was evaluated using partial AUC at 10% false positives in order to specifically prevent erroneously high-confidence false positives. The choices evaluated here include whether to assign genes to modules/pathways using MinPath (MP) or naively, inclusion of taxonomic limitation (Tax) without or with gene copy number correction (TaxC), add-one (Sm) or Witten-Bell (SmWB) abundance smoothing, and biological gap filling using pathway medians (GF) or averages (GFAve). The final HUMAnN pipeline that achieved the best overall performance is highlighted in bold and consists in the sequential execution of MinPath, taxonomic limitation with copy number correction, and median-based gap filling. (PDF) [file pcbi.1002358.s001.pdf]

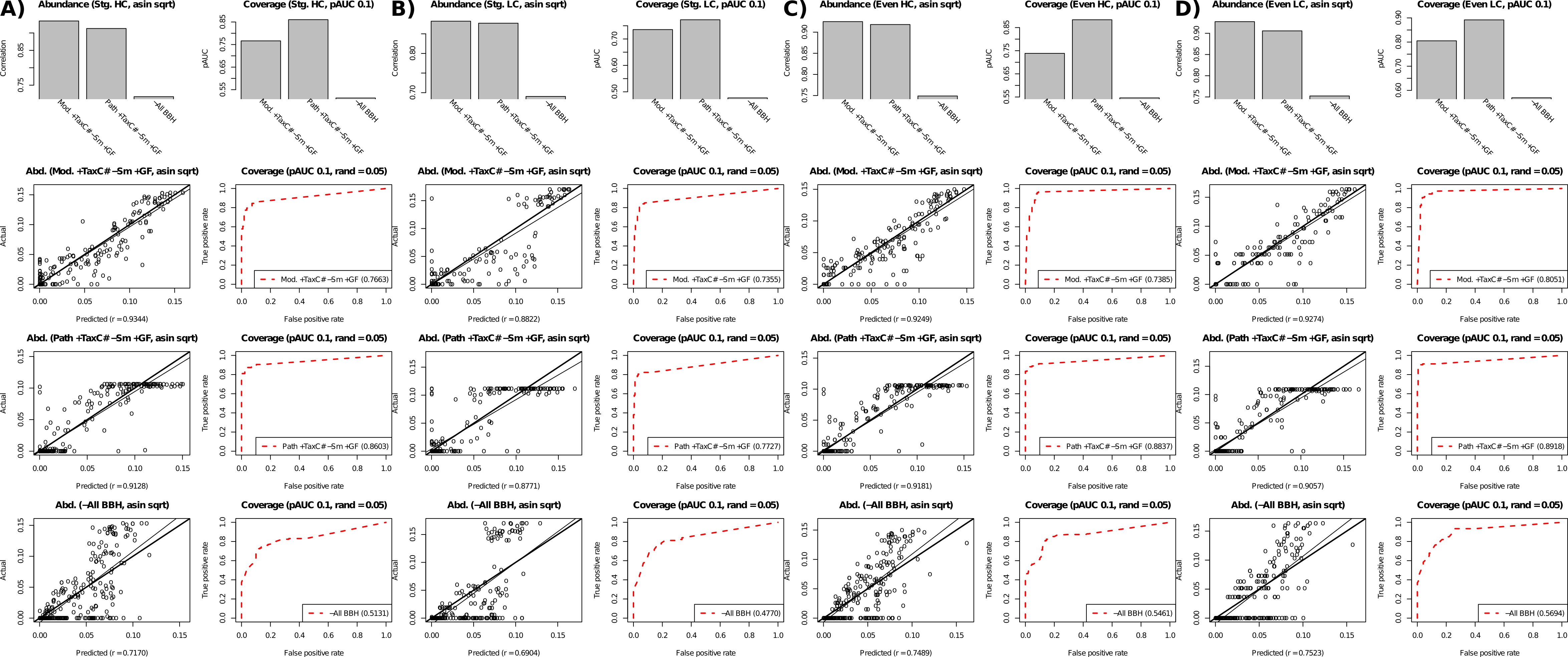

Supplement: Figure S2 — Performance of HUMAnN abundance and coverage inference as compared to a best-BLAST-hit (BBH) approach. The HUMAnN pipeline for KEGG metabolic modules and pathways was compared to a best-BLAST-hit approach to module reconstruction. Evaluations of abundance (left columns, by Pearson correlation) and coverage (right columns, by partial AUC at 10% false positives) are summarized in the first row. Subsequent rows show full scatterplots with regressions (left, abundances) and ROC curves (right, coverages) for each individual method over the entire gold synthetic community gold standards. These include A) high-complexity (100 organisms) staggered abundances B) low-complexity (20 organisms) staggered, C) high-complexity even abundances, and D) low-complexity even. (PNG) [file pcbi.1002358.s002.png]

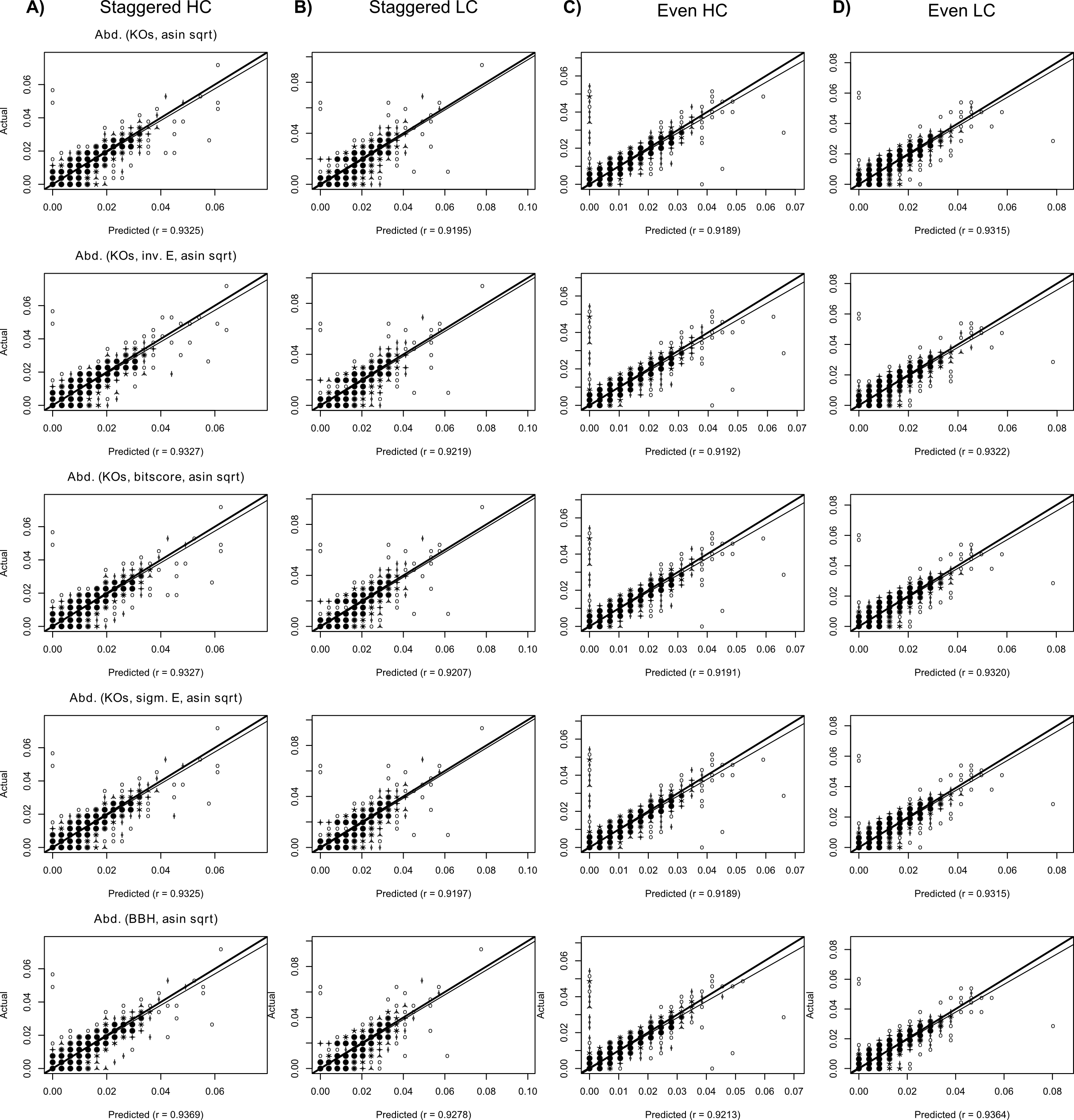

Supplement: Figure S3 — Accuracy of HUMAnN relative abundance predictions for individual KOs. Predicted versus actual abundances sunflower plotted for each of the 13,980 KEGG Orthology gene families (KOs) evaluating four E-value weighting schemes (HUMAnN's default p-value, inverse E-value, bitscore, and sigmoid E-value) and best-BLAST-hit alone in the A) the staggered high-complexity synthetic community, B) the staggered low-complexity, C) even high-complexity, and D) even low-complexity. Pearson correlation coefficients and regressions of arcsine square root transformed values are also reported. The performance of HUMAnN on the overall population of individual genes did not differ significantly from that of a best-BLAST-hit approach, although the preservation of additional information from multiple hits for later use (using any weighting scheme) does improve module and pathway abundance recovery (Supplemental Figure S2). (PNG) [file pcbi.1002358.s003.png]

Correlation (asin sqrt)

Abundance

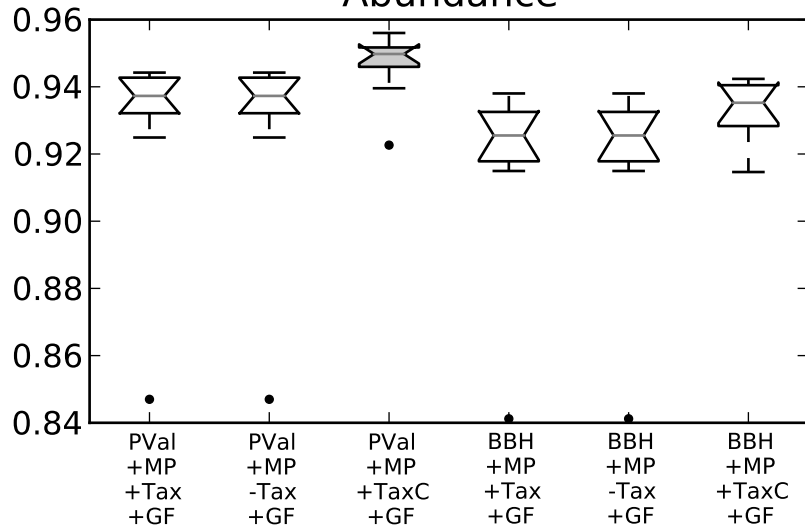

Coverage

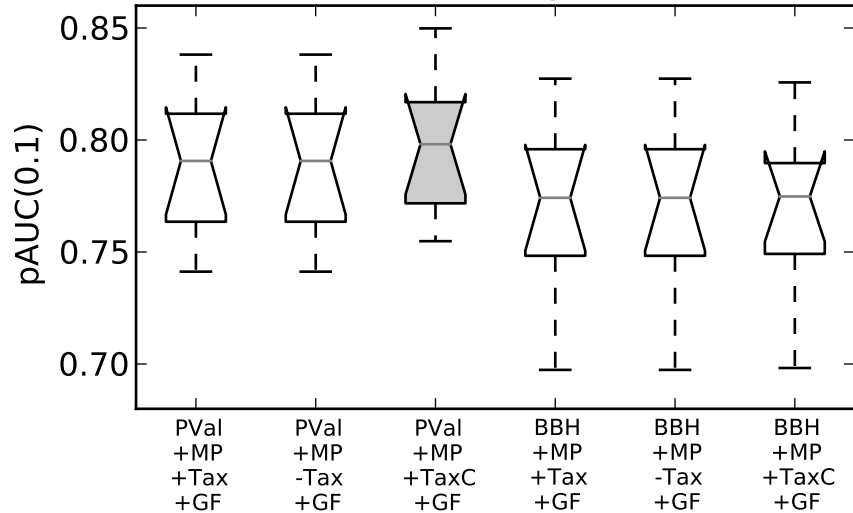

Supplement: Figure S4 — Evaluation of individual HUMAnN processing modules on 10 synthetic metagenomes. An additional 10 synthetic metagenomes were generated with high-complexity (100 organisms) and random lognormally distributed abundances. These were searched against KEGG protein sequences using USEARCH, allowing multiple hits with maximum e-value 1. All combinations of select HUMAnN modules were then assessed, including best-BLAST-hit versus multiple hits weighted by p-value and the presence or absence of taxonomic limitation with or without copy number normalization. HUMAnN default settings are highlighted in gray. Processing steps recapitulated their behavior as observed in Supplemental Figure S1. (PDF) [file pcbi.1002358.s004.pdf]

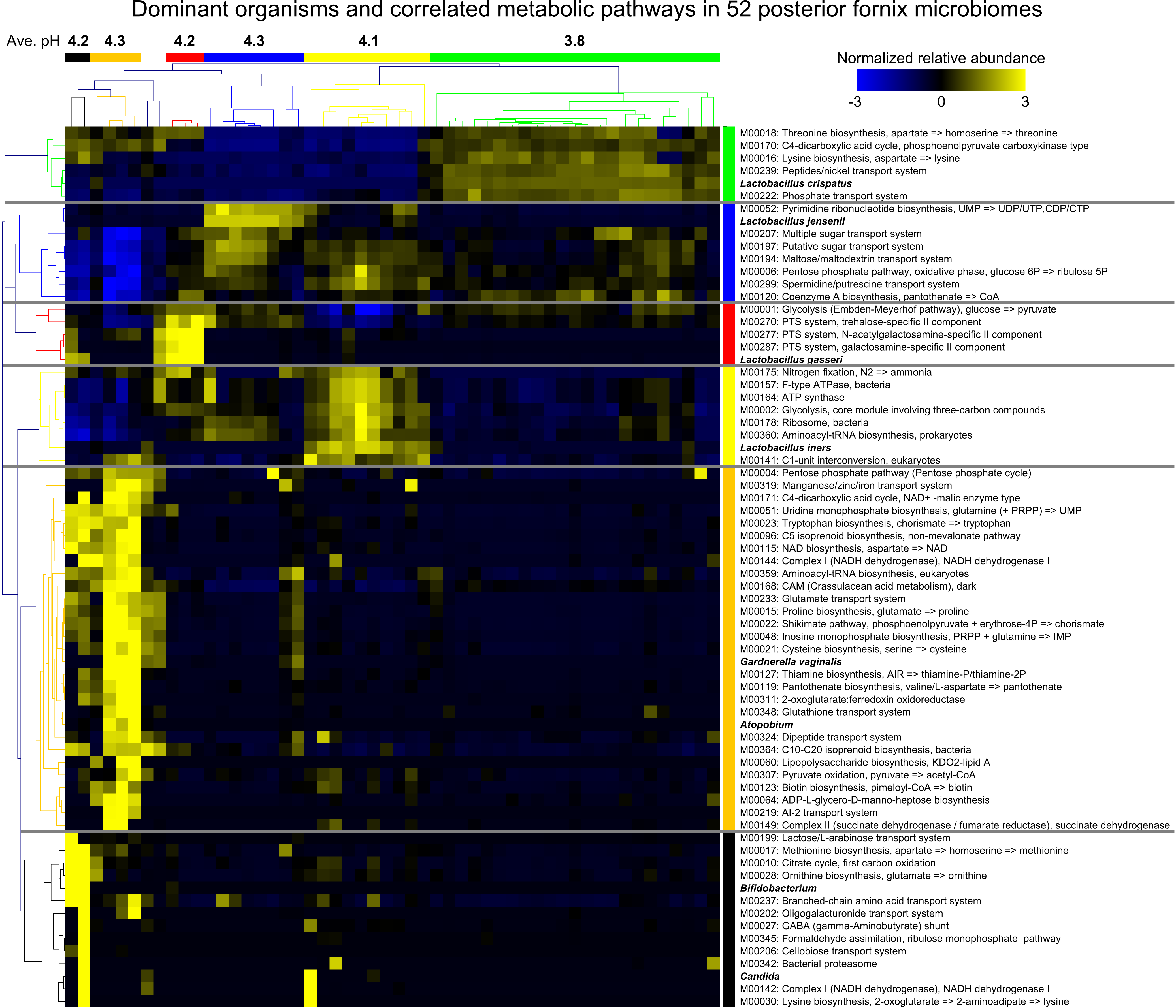

Supplement: Figure S5 — Co-clustering of metabolic pathways and microbes from community-specific genera in the vaginal microbiome. Hierarchical clustering (average linkage using uncentered Pearson correlation) was applied jointly to the eight signature organisms from the five lineages known to characterize different microbiome types [7] and to the 62 metabolic modules correlated with these taxa in the posterior fornix microbiome. All relative abundances are row z-score normalized for visualization. Differentially abundant metabolism varies directly in proportion to these signature taxa, which themselves group the 52 samples into five distinct microbiome types. These are in turn correlated with the host vaginal pH phenotype, suggesting the specialization of these organisms with respect to metabolic function in the maintenance of community structure and environmental pH. (PNG) [file pcbi.1002358.s005.png]
